# Automatic Extrinsic Calibration of 3D LIDAR and Multi-Cameras Based on Graph Optimization

**DOI:** 10.3390/s22062221

**Published:** 2022-03-13

**Authors:** Jinshun Ou, Panling Huang, Jun Zhou, Yifan Zhao, Lebin Lin

**Affiliations:** 1 School of Mechanical Engineering, Shandong University, Jinan 250061, China; oujinshun@mail.sdu.edu.cn (J.O.); zhoujun@sdu.edu.cn (J.Z.); zhaoyifan@mail.sdu.edu.cn (Y.Z.); lebin.lin@mail.sdu.edu.cn (L.L.); 2 Key Laboratory of High Efficiency and Clean Mechanical Manufacture, Ministry of Education, Jinan 250061, China

**Keywords:** automatic extrinsic calibration, graph optimization, 3D LIDAR, multi-cameras, virtual feature point

## Abstract

In recent years, multi-sensor fusion technology has made enormous progress in 3D reconstruction, surveying and mapping, autonomous driving, and other related fields, and extrinsic calibration is a necessary condition for multi-sensor fusion applications. This paper proposes a 3D LIDAR-to-camera automatic calibration framework based on graph optimization. The system can automatically identify the position of the pattern and build a set of virtual feature point clouds, and can simultaneously complete the calibration of the LIDAR and multiple cameras. To test this framework, a multi-sensor system is formed using a mobile robot equipped with LIDAR, monocular and binocular cameras, and the pairwise calibration of LIDAR with two cameras is evaluated quantitatively and qualitatively. The results show that this method can produce more accurate calibration results than the state-of-the-art method. The average error on the camera normalization plane is 0.161 mm, which outperforms existing calibration methods. Due to the introduction of graph optimization, the original point cloud is also optimized while optimizing the external parameters between the sensors, which can effectively correct the errors caused during data collection, so it is also robust to bad data.

## 1. Introduction

In recent years, with the increasing demands on perception performance in the fields of mobile robots [1,2,3], surveying and mapping [4,5,6,7,8], 3D reconstruction [9,10,11,12], and autonomous driving [13,14], the application of multi-sensor fusion technology is more and more extensive [15,16,17]. Among them, LIDAR and cameras perform particularly well in multi-sensor fusion technology [18,19,20,21]. For example, vision-based perception systems have been widely used in autonomous driving with the advantages of low cost and high performance [22,23,24]. However, the single-vision perception system cannot provide the accurate 3D information necessary for autonomous driving when acquiring 3D information [25], and the pure vision solution requires high computational cost when acquiring 3D information, and is also affected by occlusion, illumination instability, and more serious object surface texture [26]. On the contrary, LIDAR has a wide measurement range, high accuracy, and is less affected by light, but its resolution is low, and the cost is high. In order to give full play to the advantages of these two sensors, the information about the two sensors is usually fused to achieve complementary performance [20]. However, it is not easy to perfectly fuse these two sensors [27]. In addition to the performance of the sensor itself, the factors affecting the fusion effect also require accurate calibration of the external parameters of the two. Geometric calibration of extrinsic parameters is an extrinsic parameter estimation problem between two or more sensors in order to accurately correlate the 2D pixel values of camera images with real-world 3D point clouds. Due to the different methods of acquiring and storing data, the two sensors are difficult to calibrate because it is not easy to accurately match the same reference point.

As early as the 1970s, various calibration methods have appeared. For example, Heikkila and Silven [28] proposed the entire calibration process, including feature point extraction, model fitting, and image correction. Through this process, in addition to obtaining the external parameters of the two sensors, the parameters and distortion coefficients of the camera model can also be obtained at the same time, effectively compensating for image distortion. The modern calibration method also basically includes all aspects of this method. Today’s vehicle surveying and mapping equipment, mobile robots, etc., are often equipped with multiple different sensors to improve robustness and coverage. Although early calibration procedures can also meet the calibration needs of multi-sensor suites, many methods, such as Camera Calibration Toolbox for Matlab, etc. [29,30], still rely on manual extraction of feature points of laser scans, which is a time-consuming and labor-intensive process. The authors of [31] were the first to propose a simple and portable interactive lidar and camera extrinsic parameter calibration toolbox, which provides a new solution for automatic calibration of lidar-camera extrinsic parameters. Kassir and Peynot [32] proposed the first two-stage camera-to-LIDAR automatic calibration system based on the two calibration methods [29,30]. First, the system automatically calibrates the camera parameters and then performs the camera-LIDAR calibration. Geiger et al. [33] proposed a toolbox with a web interface to automatically calibrate the extrinsic parameters, making the calibration work more convenient while being robust to different lighting conditions. In [34], they used LIDAR and six cameras to form a multi-sensor system and calibrated external parameters by fitting the normals of multiple chessboards. All of the above methods use the calibration board as a reference to parameter estimation, which is an effective method. In addition to using a regular calibration board as a reference fiducial, Heng et al. [35] extended this work by using maps and natural features instead of fiducial markers. Due to the use of map features as a reference before calibrating with this method, the user must take an accurate measurement to generate a map of the calibration area. The authors of [36] used line correspondences to estimate external calibration parameters by minimizing the distance between corresponding features projected onto the image plane. In addition, the rise of deep learning has provided new ideas for calibration work. RegNet [37] was the first to use deep convolutional neural networks to estimate the 6-DOF extrinsic parameters between cameras and LIDAR. This method can provide stable initial estimates and perform continuous online corrections of external parameters. CalibNet [38] uses a geometrically supervised deep learning approach to automatically estimate the extrinsic parameters between 3D LIDAR and camera in real time, which does not require specific landmarks or scenes, nor does it requires any initial estimation of extrinsic parameters. CFNet [39] is a novel CNN-based LiDAR-camera extrinsic calibration algorithm, the method-defined calibration flow, to illustrate the deviation of the initial projection from the ground truth. It is usually necessary to collect a large number of accurately labeled data pairs for training before using deep learning methods of calibration.

The previous calibration methods have made great breakthroughs in the convenience of use, calibration accuracy, and robustness through the continuous iterative improvement of the predecessors, but most methods still rely on the measurement accuracy of the LIDAR itself. Lai and Wang [40] et al. used a constant fraction discriminator (CFD) to study the ranging distribution of LIDAR and showed that the peak position of the ranging data distribution is not affected by the signal amplitude at the optimal delay and intensity attenuation, and is close to the normal distribution. However, in the actual measurement process, it will inevitably be affected by the noise of various factors.

This paper aims to accurately solve the problem of LIDAR–camera matching difficulty due to different data structures, the sparseness of point clouds, and the noise and errors in the data acquisition process. We construct a set of virtual feature points of the LIDAR reference frame of pairwise matching with the feature points in a camera image. Then, we use the group of virtual feature points as the optimized initial value, establish the least square constraint on the spatial feature points and the external parameters of the two sensors, and construct the graph model. Finally, the method of graph optimization is used to optimize the spatial coordinate of the feature points and the external parameters between sensors at the same time. This method has low requirements for data collection accuracy, all feature points are involved in optimization, and the accuracy of the calculation result is high.

The main contribution of this paper is to use the laser reflectivity to make the LIDAR automatically identify and locate the position of the calibration board and to construct a set of virtual feature corner points according to the position of the calibration board to match the feature points in the image pixels. This avoids the need for manual matching of feature points in methods such as [29,30,31]. The virtual feature points obtained by this method after statistical analysis can be used as the initial value for optimization. Second, most robotic systems have more than two sensors to be calibrated, and the use of pairwise calibration in such systems will rapidly increase the number of sensor combinations to be calibrated, such as [34,41]. In this paper, the problem of external parameter calibration of the LIDAR-camera system is solved by means of graph optimization, and the external parameters of the LIDAR-camera and the position of LIDAR feature points are taken as the vertices of the graph model. In principle, we can insert as many vertices as external parameters to be optimized to remove limitations of the number of LIDAR-camera external parameter calibrations. Finally, due to the introduction of the graphical model, the feature points of the LIDAR measurement will also be optimized while optimizing the external parameters, which can effectively solve the error problem of the LIDAR measurement process, while many calibration methods only focus on the optimization of external parameters. Therefore, the method proposed in this paper can generally be used in multi-sensor systems that require simultaneous calibration of LIDAR and multiple cameras and in situations where the confidence of LIDAR measurements is low.

This work is organized as follows. The next section firstly details the automatic extraction and registration method of laser point cloud and image feature points and analyzes the feasibility of the feature point extraction method. Then, we introduce the LIDAR-camera extrinsic parameter estimation and graph optimization methods and summarizes the algorithms of the entire calibration process. Section 3 conducts experiments on the method proposed in this paper. Section 4 discusses the experimental results and compares them with several commonly used methods; the paper is concluded with Section 5.

## 2. The Proposed Method

This section firstly introduces the autonomous identification and registration methods of LIDAR and image feature points and analyzes its feasibility. Then, we describe the proposed graph optimization framework and introduce virtual feature points as the initial optimization value to replace the real feature points that are difficult to measure so that they can participate in the optimization together with external parameters. As an example, we study how a three-sensor system can perform the calibration of two pairs of sensors simultaneously using 60 sets of raw data. In this process, we use a large number of symbols and functions to represent and calculate the position of the same feature point in different sensor coordinates systems, as well as the external parameters between different sensors. These symbols and functions are explained in detail in Section 2.1 and Section 2.2. The algorithm pseudocode of the entire calibration process is summarized at the end of Section 2.2.

### 2.1. Automatic Registration of LIDAR Point Cloud and Image Feature Points

#### 2.1.1. Autonomous Identification Calibration Board

**Figure 1:**
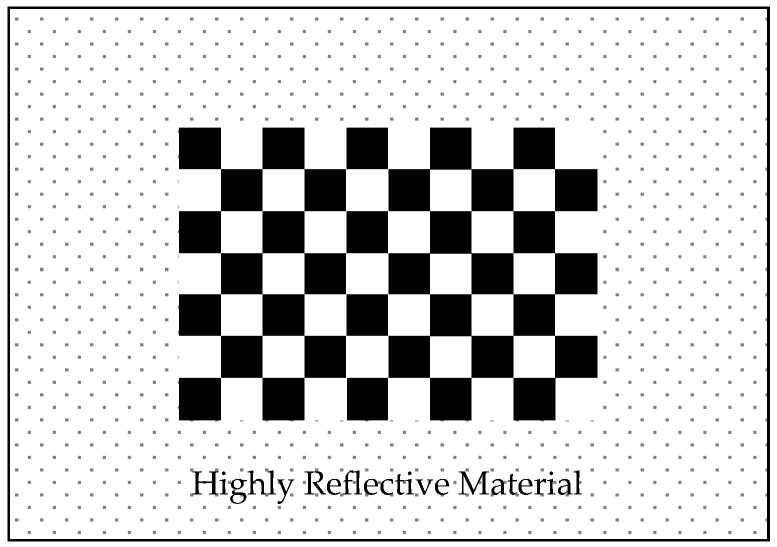
Autonomous identification calibration board.

According to the intensity characteristics of the LIDAR, we designed a calibration board that can be automatically recognized by the LIDAR and camera at the same time, which provides a basis for the automatic calibration of LIDAR and camera external parameters. The calibration board is improved on the basis of the checkerboard calibration board proposed by Zhang [42]. The calibration board adopts a 10 × 7 checkerboard, and the edge of the checkerboard is equipped with highly reflective materials, as shown in Figure 1.

The internal checkerboard of the calibration board provides 9 × 6 characteristic corner points for the camera. When the LIDAR scans the external high-reflective material, it gets intensity information that is higher than the scanning point of the surrounding environment. According to this feature, the camera and LIDAR can automatically identify the feature point information and point cloud information of the calibration board at the same time.

#### 2.1.2. Automatic Registration of Point Cloud and Image

1. Point Cloud Processing

**Figure 2:**
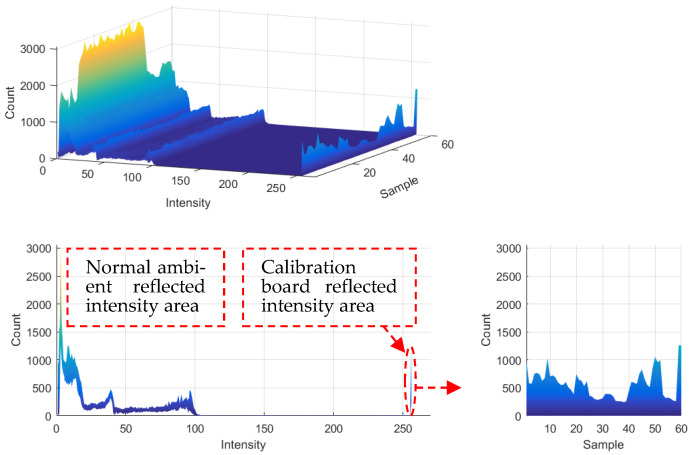
Statistics of the intensity of laser scanning reflector and surrounding environment.

Figure 2 counts the intensity of 60 groups of LIDAR systems when scanning the reflector and surrounding environment. The red ellipse-marked area is the point cloud statistics when the laser irradiates the reflector. Because of the divergence of the laser, the number of point clouds on the calibration plate differs at different distances. However, it is found that LIDAR still has a good difference in intensity between the ordinary environment and reflector. Therefore, the intensity threshold is set to 250, based on the intensity information that filters out all the point clouds on the reflector, and each point in the point cloud contains the laser beam id.

For each group of point clouds filtered from the raw point cloud, we use the following steps to estimate the spatial position of the calibration board and linearly fit the initial coordinates of the 9 × 6 checkerboard corner points on the calibration board.

**Figure 3:**
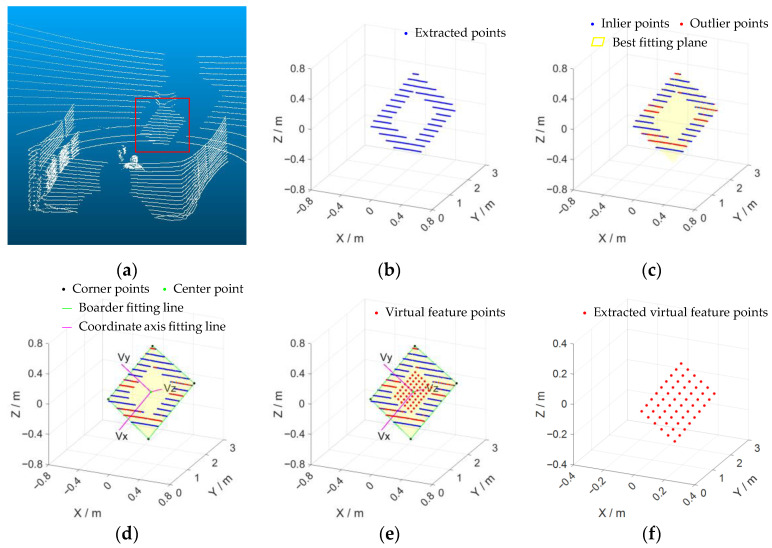
Virtual feature corner extraction process, from (**a**–**f**). (**a**) Original point cloud; (**b**) extract the point cloud on the calibration board according to the intensity; (**c**) fit the plane of the calibration board; (**d**) fit the edge of the calibration board, and determine the calibration board coordinate system; (**e**) fit virtual feature corner points; (**f**) get virtual feature corner points.

Using the random sampling consensus (RANSAC) [43] algorithm, the point cloud ${\hat{P}}^{L}$ is used to fit the plane where the calibration plate is located, as shown in Figure 3c.

According to the id information, we select the starting point ${\hat{p}}_{id}^{l}$ and the ending point ${\hat{p}}_{id}^{r}$ in the point cloud corresponding to each laser scan line id in ${\hat{P}}^{L}$, and project all ${\hat{p}}_{id}^{l}$ and ${\hat{p}}_{id}^{r}$ to the fitting plane. The projection points are $p_{id}^{l}$ and $p_{id}^{r}$, respectively, using the RANSAC line fitting algorithm and $\left(p_{id}^{l},p_{id}^{r}\right)$, we fit the four sides of the calibration board, and the direction vector is expressed as $n=(n_{x0},n_{x1},n_{y0},n_{y1})$. The four sides intersect in pairs to find the four corner points of the calibration board, expressed by $P_{corner}^{L}=(p_{0},p_{1},p_{2},p_{3})$.

The average value of $P_{corner}^{L}$ is calculated as the center of the calibration board $P_{center}^{L}$, and the average value of n is calculated as the unit direction vector of the two coordinate axes of the calibration board coordinate system, expressed by $n_{x}$, $n_{y}$.

**Figure 4:**
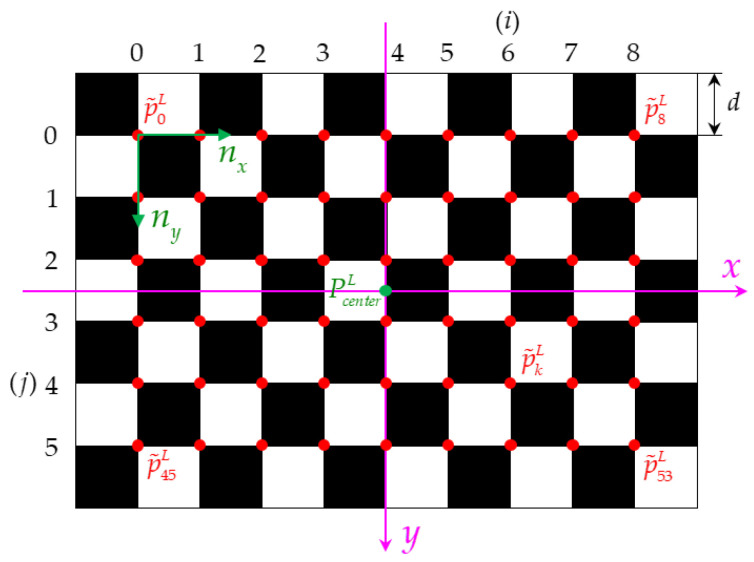
Schematic diagram of the position of each corner point in the LIDAR coordinate system.

According to the side length d of the checkerboard and $P_{center}^{L}$, $n_{x}$, $n_{y}$, we create the initial coordinates of the virtual feature corners ${\tilde{P}}^{L}=({\tilde{p}}_{0}^{L},\ldots ,{\tilde{p}}_{k}^{L},\ldots ,{\tilde{p}}_{53}^{L})$, as shown in Figure 4, and define the position of each corner point in the LIDAR coordinate system as:

(1) $${\tilde{p}}_{k}^{L}=P_{center}^{L}-(4-i)d\cdot n_{x}-(2.5-j)d\cdot n_{y}\;\left(k=0,1,\ldots ,53;\;i=0,1,\ldots ,8;\;j=0,1,\ldots ,5\right)$$

The extracted virtual feature corners are shown in Figure 3f.

2. Image Feature Point Extraction

**Figure 5:**
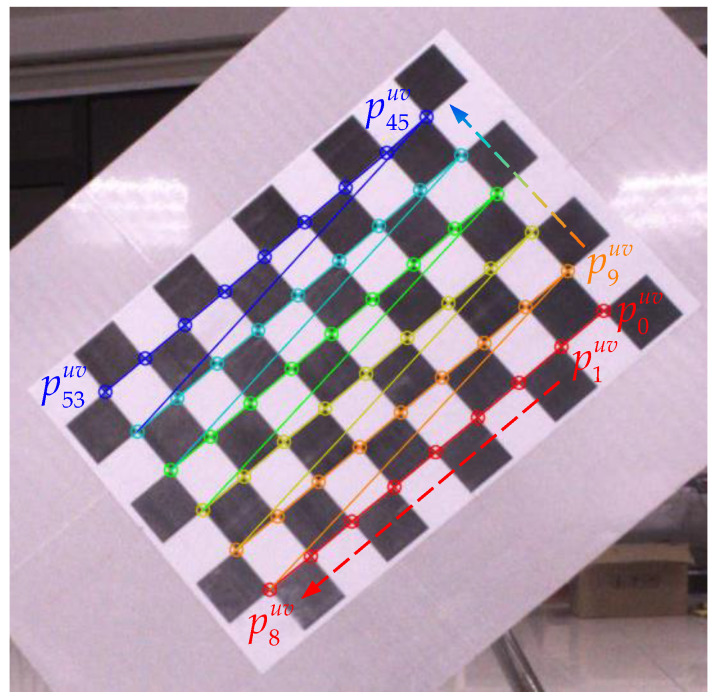
Subpixel checkerboard corner detection.

The calibration precision also depends on the precise location of the image feature corner at the sub-pixel level [44]. Therefore, for the feature points in the image, we use the sub-pixel-level corner detection method provided by OpenCV [45] to extract the pixel coordinates of the 9 × 6 corners of the checkerboard, expressed by $P_{}^{uv}=(p_{0}^{uv},p_{1}^{uv},\ldots ,p_{53}^{uv})$, as shown in Figure 5.

### 2.2. Extrinsic Calibration Algorithm Based on Graph Optimization

The coordinates of the characteristic corners of the checkerboard in the LIDAR coordinates system are estimated by the fitting plane and fitting edge of the calibration board. In this process, the measurement noise and estimation error of the LIDAR is inevitably included. Therefore, when estimating the extrinsic parameters of the LIDAR and camera, the error of extrinsic parameter estimation is also included. Aiming at this series of noise and errors, this paper uses graph optimization to optimize the spatial coordinates of the feature corners and the external parameters between the camera and LIDAR at the same time.

#### 2.2.1. Constructing the Least Squares Problem

For a point ${\tilde{p}}^{L}$ in the LIDAR coordinate system and its observation value $p^{uv}$ on the camera pixel plane, we aimed to determine the external parameters between the LIDAR and the camera. We used the Lie group SE(3) to represent the external parameters between the LIDAR and the camera [46]; Lie algebra is represented as ξ. When the camera’s internal parameter K and the distortion coefficient $\left(k_{1},k_{2}\right)$ has been accurately calibrated, we can use the external parameters, the pinhole camera model, and the distortion model to calculate the projection ${\tilde{p}}^{uv}$ of ${\tilde{p}}^{L}$ on the pixel plane. Because the tangential distortion is very small, it is not considered for the time being. The process is as follows.

Firstly, according to the external parameters between the LIDAR and the camera, the spatial point of the LIDAR coordinate system in the camera coordinate system is:

(2) p˜C=[p˜xC,p˜yC,p˜zC]T=exp(ξ^)p˜L

Then, we project the ${\tilde{p}}^{C}$ to the normalized plane as:

(3) $$p_{norm}^{C}={\left[p_{norm\_x}^{C},p_{norm\_y}^{C},1\right]}^{T}={\tilde{p}}^{C}/{\tilde{p}}_{z}^{C}$$

According to the radial distortion model [47], the projected point coordinates of this point on the normalized plane after radial image distortion is:

(4) $$p_{dist}^{C}={\left[p_{dist\_x}^{C},p_{dist\_y}^{C},1\right]}^{T}=(1+k_{1}r_{c}^{2}+k_{2}r_{c}^{4}){\left[p_{norm\_x}^{C},p_{norm\_y}^{C},1+k_{1}r_{c}^{2}+k_{2}r_{c}^{4}\right]}^{T}$$

where $r_{c}^{2}={\left(p_{norm\_x}^{C}\right)}^{2}+{\left(p_{norm\_y}^{C}\right)}^{2}$.

Finally, according to the internal camera parameter K, the pixel coordinates of this point on the camera imaging plane is:

(5) $${\tilde{p}}^{uv}=Kp_{dist}^{C}$$

**Figure 6:**
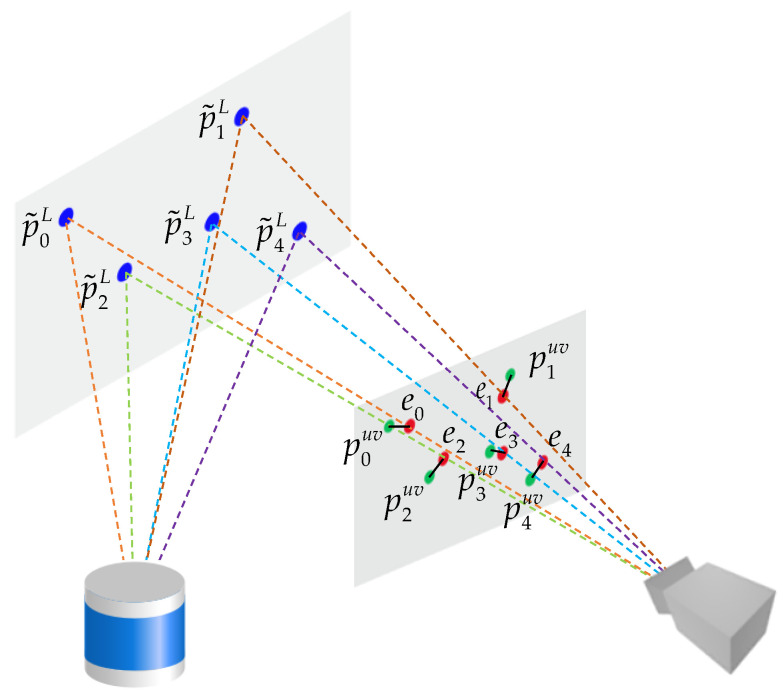
Re-projection error of characteristic corner points.

The precise external parameters of the LIDAR and the camera are unknown, and there are noises in the observations of the LIDAR and the camera. Therefore, there is an error e between the characteristic corner coordinates ${\tilde{p}}^{uv}$ calculated by the model, denoted as $h(\xi ,{\tilde{p}}^{L})$, and the actual observed corner coordinates $p^{uv}$, denoted as z. As shown in Figure 6, where the blue point ${\tilde{p}}_{i}^{L}$ represents the spatial corner point observed by LIDAR, the green point $p_{i}^{uv}$ represents the corner point observed by the camera, the red point represents the projection of the spatial corner point on the pixel plane, and the black solid line $e_{i}$ represents the re-projection error, it can be expressed as:

(6) $$e=z-h(\xi ,{\tilde{p}}^{L})$$

We construct the least-squares problem by accumulating all the errors and use $e_{i}$ to represent the error produced by observing the *i*-th feature point, then the total error loss generated by the entire observation process is:

(7) $$ℒ=\frac{1}{2}\sum_{i=0}^{n-1}{\left\|e_{i}\right\|}^{2}=\frac{1}{2}\sum_{i=0}^{n-1}{\left\|z_{i}-h(\xi ,{\tilde{p}}_{i}^{L})\right\|}^{2}$$

Solving this least-squares problem, the external parameter ξ and the spatial position ${\tilde{P}}^{L}$ of the corner points can be optimized at the same time. The optimization results are:

(8) $$(\xi *,p_{i}^{L})=\arg\underset{\xi ,{\tilde{p}}_{i}^{L}}{\min}\frac{1}{2}\sum_{i=0}^{n-1}{\left\|z_{i}-h(\xi ,{\tilde{p}}_{i}^{L})\right\|}^{2}$$

#### 2.2.2. Graph Optimization Model and Solution

When solving the above least squares problem, we constructed an unconstrained optimization model by using Lie algebra. Since this is a non-linear optimization model, it is not convenient to find the optimal value directly by finding the zero solution of the derivative of the loss function. Therefore, we use the gradient descent method to continuously update the optimization variables to reduce the value of the loss function.

We denote the optimized variable by x. In the process of updating the optimized variable x, each iteration selects an appropriate increment Δx in the opposite direction of the gradient to reduce the loss function. When Δx is small enough, the loss function does not continue to decrease so as to reach the minimum value. At this time, the optimized variable is the optimal solution. Therefore, choosing an appropriate increment Δx is the key to finding the optimal solution. Commonly used methods are the Newton iteration method, Gauss–Newton [48], and Levenberg–Marquardt iteration [49] method. Here, we used the Gauss–Newton iteration method to construct the incremental equation of this least-squares problem.

Representing the loss as a function f(x) with respect to x:

(9) $$ℒ=\frac{1}{2}{\left\|f(x)\right\|}^{2}$$

Perform a first-order Taylor expansion of f(x) near x, and the loss function is:

(10) $$ℒ=\frac{1}{2}{\left\|f(x+\Delta x)\right\|}^{2}\approx \frac{1}{2}{\left\|f(x)+J(x)\Delta x\right\|}^{2}$$

The Jacobian matrix J(x) is the derivative of f(x) with respect to x.

For an optimized variable x in a certain state, the increment Δx that minimizes the loss function is:

(11) $$\Delta x*=\arg\underset{\Delta x}{\min}\frac{1}{2}{\left\|f(x)+J(x)\Delta x\right\|}^{2}$$

The loss function takes the derivative of Δx and sets it to zero to obtain the incremental equation [50]:

(12) HΔx=g

where $H=J{\left(x\right)}^{T}J(x),g=-J{\left(x\right)}^{T}f(x)$. The above equation is also known as the Gauss–Newton equation or Normal equation. Here, H uses $J^{T}J$ to approximately replace the second-order Hessian matrix in Newton’s method, which can greatly improve the calculation efficiency.

For our joint calibration problem, the optimization variable x includes two external parameters represented by Lie algebra ξ, which is a six-dimensional vector and includes n 3D points. Representing x as a vector,$x={\left[\xi_{1},\xi_{2},{\tilde{p}}_{0}^{L},{\tilde{p}}_{1}^{L},\ldots ,{\tilde{p}}_{n-1}^{L}\right]}^{T}$.

Then, the increment of the corresponding optimization variable is:

$$\Delta x={\left[\Delta \xi ,\Delta p^{L}\right]}^{T}={\left[\Delta \xi_{1},\Delta \xi_{2},\Delta {\tilde{p}}_{0}^{L},\Delta {\tilde{p}}_{1}^{L},\ldots ,\Delta {\tilde{p}}_{n-1}^{L}\right]}^{T}$$

When the loss function updates an increment Δx.

(13) $$ℒ=\frac{1}{2}{\left\|f(x+\Delta x)\right\|}^{2}\approx \frac{1}{2}\sum_{i=1}^{2}\sum_{j=1}^{n}{\left\|e_{ij}+F_{ij}\Delta \xi_{i}+E_{ij}\Delta {\tilde{p}}_{j}^{L}\right\|}^{2}$$

The matrix $F_{ij}$ is the partial derivative of the loss function with respect to the external parameter ξ, and $E_{ij}$ is the partial derivative of the loss function with respect to the corner point ${\tilde{p}}_{j}^{L}$.

Expressing the above formula for the overall form, that is, taking the derivative of the overall optimization variable:

(14) $$ℒ=\frac{1}{2}{\left\|f(x+\Delta x)\right\|}^{2}=\frac{1}{2}{\left\|e+F\Delta \xi +E\Delta {\tilde{p}}_{}^{L}\right\|}^{2}$$

The Jacobian matrix J and the incremental equation H matrix of the above formula are:

(15) $$J=\left[\begin{array}{cc} F & E \end{array}\right]$$

(16) $$H=J^{T}J=\left[\begin{array}{cc} F^{T}F & F^{T}E\\ E^{T}F & E^{T}E \end{array}\right]$$

Because J is obtained by taking partial derivatives of all corner points and poses, and the number of corner points that need to be optimized is huge, the dimension of the H matrix is very large, and it is extremely inconvenient to directly solve the incremental equation by inverting H. However, fortunately, the H matrix is a sparse matrix; therefore, using the special structure of the matrix, we can easily solve this incremental equation and use graph optimization to express it. Graph optimization [51] is a commonly used optimization method in visual slam because, when solving bundle adjustment (BA) [52] problems in visual slam, a large number of feature points and camera poses need to be processed, which lead to a huge amount of calculations in BA. Manolis Lourakis et al. [53] found that the BA problem is actually sparse. It is by using this feature that visual slam based on graph optimization can be realized. The problem faced in this paper is similar to the slam problem and involves much less marking points and sensor poses. Therefore, using this method can optimize the position of feature points and poses between multiple sensors at the same time, and we take into account the accuracy of the feature points of the calibration plate fitted. In view of this, it is feasible to use the graph optimization method to calibrate external parameters between LIDAR and multiple cameras.

**Figure 7:**
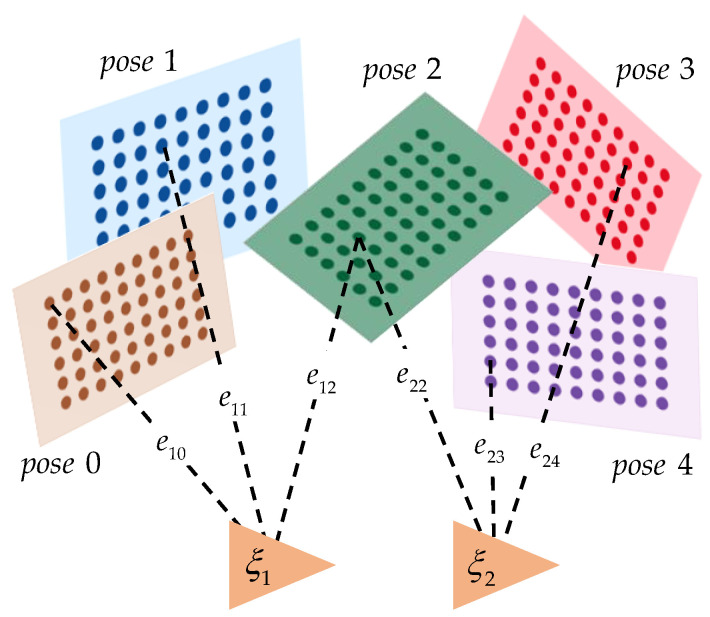
Joint calibration graph optimization model.

Next, we build a graph model composed of external parameters between LIDAR and camera and all corner points, as shown in Figure 7. The triangles represent the external parameters, the rectangles represent the calibration boards in different poses, the solid points represent the characteristic corners, and the dotted lines represent the errors caused by observing the corners under the current external parameters.

According to the graph model, the loss function of the entire observation is:

(17) $$ℒ=\frac{1}{2}({\left\|e_{10}\right\|}^{2}+{\left\|e_{11}\right\|}^{2}+{\left\|e_{12}\right\|}^{2}+{\left\|e_{22}\right\|}^{2}+{\left\|e_{23}\right\|}^{2}+{\left\|e_{24}\right\|}^{2})$$

The Jacobian matrix J of the loss function and its corresponding H matrix are:

(18) $$J={\left[\begin{array}{cccccc} J_{10} & J_{11} & J_{12} & J_{22} & J_{23} & J_{24} \end{array}\right]}^{T}=\left[\begin{array}{cc} \frac{\partial ℒ}{\partial \xi_{1}} & \frac{\partial ℒ}{\partial {\tilde{p}}_{i}^{L}}\\ \frac{\partial ℒ}{\partial \xi_{2}} & \frac{\partial ℒ}{\partial {\tilde{p}}_{j}^{L}} \end{array}\right]$$

In this example, i,j=0,…,4. In the actual calibration process, the number of points is up to thousands, and the external parameters can be at least one or more.

(19) $$H=J^{T}J=\left[\begin{array}{cc} {\left(\frac{\partial ℒ}{\partial \xi_{1}}\right)}^{T}\frac{\partial ℒ}{\partial \xi_{1}}+{\left(\frac{\partial ℒ}{\partial \xi_{2}}\right)}^{T}\frac{\partial ℒ}{\partial \xi_{2}} & {\left(\frac{\partial ℒ}{\partial \xi_{1}}\right)}^{T}\frac{\partial ℒ}{\partial {\tilde{p}}_{i}^{L}}+{\left(\frac{\partial ℒ}{\partial \xi_{2}}\right)}^{T}\frac{\partial ℒ}{\partial {\tilde{p}}_{j}^{L}}\\ {\left(\frac{\partial ℒ}{\partial {\tilde{p}}_{i}^{L}}\right)}^{T}\frac{\partial ℒ}{\partial \xi_{1}}+{\left(\frac{\partial ℒ}{\partial {\tilde{p}}_{j}^{L}}\right)}^{T}\frac{\partial ℒ}{\partial \xi_{2}} & {\left(\frac{\partial ℒ}{\partial {\tilde{p}}_{i}^{L}}\right)}^{T}\frac{\partial ℒ}{\partial {\tilde{p}}_{i}^{L}}+{\left(\frac{\partial ℒ}{\partial {\tilde{p}}_{j}^{L}}\right)}^{T}\frac{\partial ℒ}{\partial {\tilde{p}}_{j}^{L}} \end{array}\right]$$

Using a block matrix to represent the above matrix:

(20) $$H=\left[\begin{array}{cc} B & E\\ E^{T} & C \end{array}\right]$$

Then the incremental equation is:

(21) $$\left[\begin{array}{cc} B & E\\ E^{T} & C \end{array}\right]\left[\begin{array}{c} \Delta \xi \\ \Delta p^{L} \end{array}\right]=\left[\begin{array}{c} a\\ b \end{array}\right]$$

Obviously, C is a diagonal matrix, so it is easy to invert. Solving the above formula, we get:

(22) $$\Delta \xi ={\left[B-EC^{-1}E^{T}\right]}^{-1}\left[a-EC^{-1}b\right]$$

(23) $$\Delta p^{L}=C^{-1}(b-E^{T}\Delta \xi )$$

We summarize our algorithm as shown in Algorithm 1.
**Algorithm 1** Graph Optimization Calibration of LIDAR-Cameras**Input:** Source point cloud: $P_{src}^{L}$; Source image: Img; Camera internal parameters and distortion coefficient: $K,dist=[k_{1},k_{2}]$.1:// Extract the point cloud of the calibration board:2:**for** $p_{src\_i}^{L}$ in $P_{src}^{L}$ **do**3:   **for** p in $p_{src\_i}^{L}$ **do**4:    **if** intensity>threshold **then**5:     add $p\to {\hat{P}}_{i}^{L}$;6:    **end if**
7:  **end for**
8:**end for**9:// Virtual feature point fitting:10:**for** each ${\hat{P}}_{i}^{L}$ **do**11:   Plane fitting: ${\hat{P}}_{i}^{L}\overset{RANSAC}{\to }\alpha$;12:   Filter left and right edge points: ${\hat{P}}_{i}^{L}\overset{ID}{\to }{\hat{P}}_{l}^{L},{\hat{P}}_{r}^{L}$;13:   Project edge points to α: ${\hat{P}}_{l}^{L},{\hat{P}}_{r}^{L}\to {\hat{P}}_{l\_proj}^{L},{\hat{P}}_{r\_proj}^{L}$;14:   Fit four edge lines: ${\hat{P}}_{l\_proj}^{L},{\hat{P}}_{r\_proj}^{L}\overset{RANSAC}{\to }l_{l0},l_{l1},l_{r0},l_{r1}$;15:   Calculate the center point and coordinate axis of the calibration board: $l_{l0},l_{l1},l_{r0},l_{r1}\to p_{center}^{L},n_{x},n_{y}$;16:   Calculate virtual feature corners: $p_{center}^{L},n_{x},n_{y}\to {\tilde{P}}_{i}^{L}$, (Formula (1));17:**end for**18:// Extract image feature points:19:**for** $img_{i}$ in Img **do**20:   Subpixel corner point extraction: $img_{i}\to P_{i}^{uv}$;21:**end for**22:3D-2D feature point matching: $\left({\tilde{P}}_{i}^{L},{\tilde{P}}_{i}^{uv}\right)$;23:Use EPnP algorithm to solve ξ: $({\tilde{P}}_{i}^{L},{\tilde{P}}_{i}^{uv})\overset{EPnP}{\to }\xi$;24:// Graph optimization to solve external parameters:25:Set the initial value: $x=[\xi ,{\tilde{P}}^{L}]$;26:**while** True **do**27:   Calculate the Jacobian matrix $J(x_{k})$ and the error $f(x_{k})$, (Formulas (18) and (9));28:   Calculate the increment equation: $\Delta x_{k}=[\Delta \xi ,\Delta P^{L}]$, (Formulas (22) and (23));29:   **if** $\Delta x_{k}>threshold$ **then**30:    $x_{k+1}=x_{k}+\Delta x_{k}$;31:   **else**
32:    return $\xi *,P^{L}$& break;33:   **end if**
34:**end while****Output:** LIDAR and cameras external parameter: ξ*; Optimized feature points $P^{L}$.

## 3. Experiment

In order to verify the calibration method and effect of this paper, we conducted experiments on the abovementioned theories. First, a calibration board was made according to the above method, and the internal parameters and distortion coefficient of the camera were calibrated. Then, we completed the construction of the LIDAR and camera physical platform and collected the original data. Finally, the data were processed according to the graph optimization method, and the final results were analyzed.

### 3.1. Experimental System Construction

**Figure 8:**
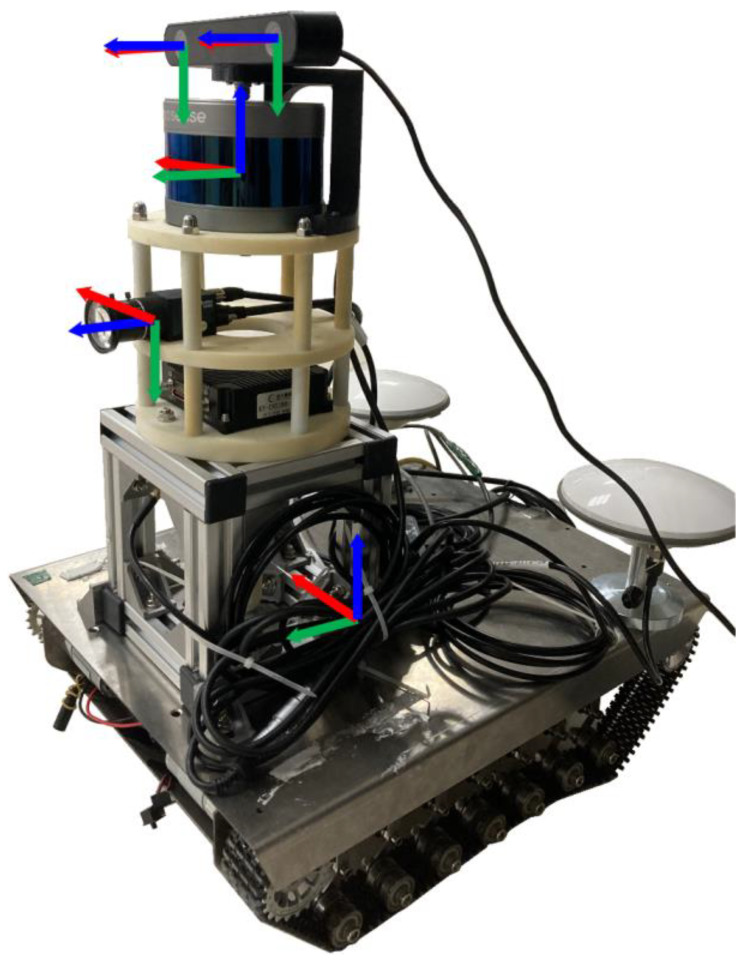
Autonomous mobile robot and the layout of each sensor, the frame of each sensor reference system is represented as a red-green-blue axes.

Our data acquisition system used RS-LIDAR-16 LIDAR, MER-132-43GC-P camera, and ZED2 camera (use only left eye), and the relative poses of the three were kept constant during calibration, as shown in Figure 8. The main parameters are shown in Table 1 and Table 2.

The inside of the calibration board adopted a 10 × 7 checkerboard pattern. The side length of each square was 55 mm, the total size was 385 mm × 550 mm, and the total size of the peripheral high reflection area was 1000 mm × 700 mm, which was also the total size of the entire calibration board.

### 3.2. Data Collection and Processing

During data collection, the positions of the LIDAR and the camera were kept fixed, and the calibration board was placed obliquely to ensure that as many LIDAR scan lines as possible passed through the edges of the calibration board. In order to increase the generality of data collection, in addition to making the calibration board face the LIDAR and camera, it can also be rotated around the vertical direction at a certain angle so that the calibration plate is biased towards the LIDAR and camera. The inclination between the plane of the calibration board and the ground can also be appropriately increased, but it is still necessary to ensure that the laser scanning lines pass through the edge of the calibration board as much as possible. When collecting each group of data, only the calibration board was moved, and the moving range was kept within 2-5 m from the LIDAR and the camera, while ensuring that the camera and LIDAR observed the entire calibration board. LIDAR point cloud data and camera image data were collected once at each location, and a total of 60 sets of data were collected.

**Figure 9:**
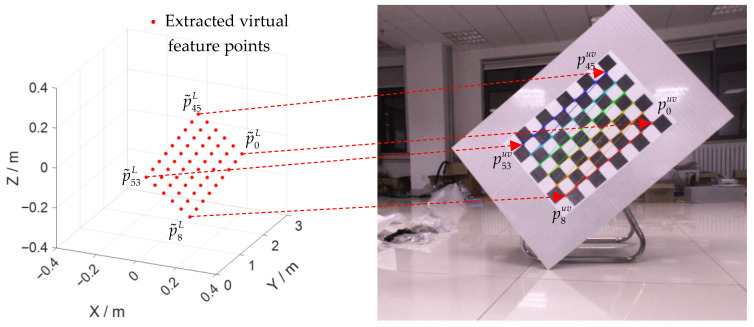
Correspondence between spatial corner points and pixel corner points.

For each set of data, according to the method proposed in this paper, we fit the coordinate ${\tilde{P}}^{L}$ of the characteristic corner point in the LIDAR coordinate system and the coordinate $P^{uv}$ in the camera pixel coordinate system and made them correspond to each other, as shown in Figure 9. Combining all the data together, the 3D points ${\tilde{P}}^{L}$ form a 3240 × 3 matrix, and the 2D points $P^{uv}$ are a 3240 × 2 matrix.

Then, using the EPnP [54] algorithm to estimate the external parameters ξ of LIDAR and camera by matching 3D-2D feature corner points and using this result as the initial value of graph optimization. Finally, we input all the 3D-2D point pairs and initial values of the extrinsic parameters into the graph optimization model and calculated the optimized 3D point cloud and extrinsic parameters.

## 4. Results and Discussion

**Figure 10:**
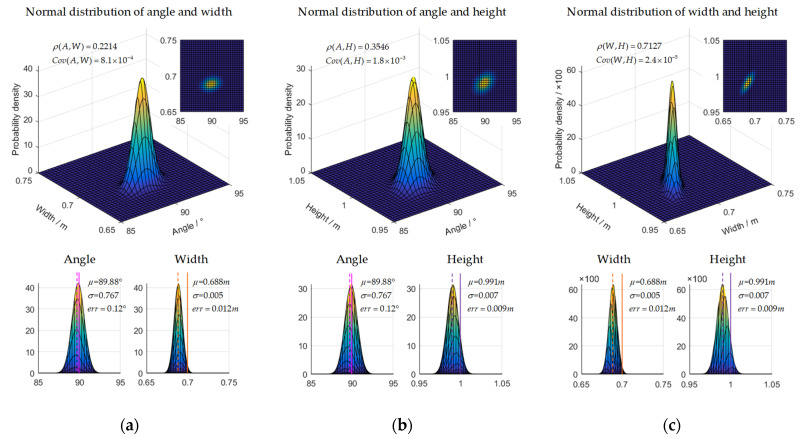
The size fitting analysis of each side and angle of the calibration board, the solid line represents the true value, and the dashed line represents the fitted value. (**a**) Fitting distribution of angle and width. (**b**) Fitting distribution of angle and height. (**c**) Fitting distribution of width and height.

We counted the results of each group of the point cloud fitting calibration board. The joint distribution of each parameter is shown in Figure 10.

As the LIDAR scanned the edge of the calibration board, there was a weaker scanning area, about 0.005 m at each edge, so the final fitted side length was 0.01 m smaller than the actual side length; the statistical results support this. In addition, according to the statistical results, the correlation between the angle and side length was small, so even if the side length error was large, a more accurate angle could be obtained, and the fitting error was only 0.12°. Because each edge shrunk uniformly, the center position and angle of the final fitting were basically unchanged. Therefore, according to the size error between the fitting results and the actual calibration board, it is shown that the spatial coordinates of the characteristic corner points fitted by the original point cloud can provide a good initial value of the point cloud for the graph optimization model.

**Figure 11:**
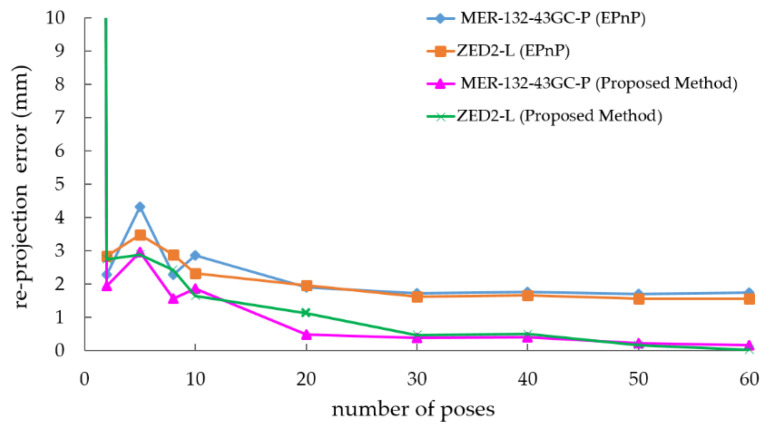
Re-projection error of normalized plane with different number of poses.

For the two sensors to be calibrated, as shown in Figure 11, the error was relatively large and unstable at the beginning. When more poses were added to participate in the optimization, the error converged quickly and became stable at 20 frames. The average re-projection error of the sensor in the normalized plane gradually decreased to less than 1 mm. Compared with methods without graph optimization, our method showed superior performance after 10 frames. As the number of poses involved in the optimization further increased, the accuracy of the optimization results still increased with a slight trend, eventually reaching a high level.

**Figure 12:**
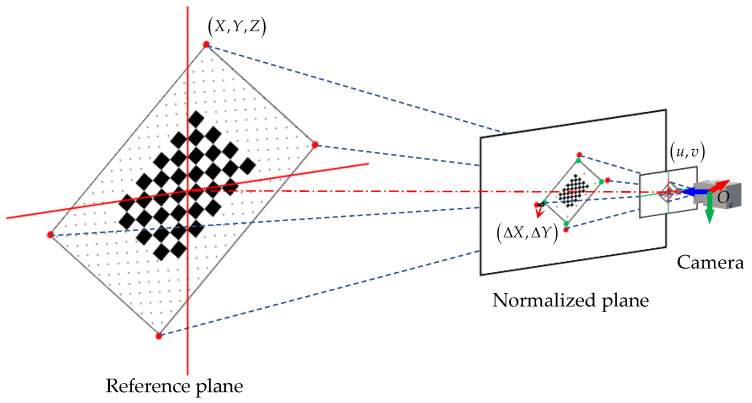
Schematic diagram of the re-projection error of the normalized plane.

Table 3 and Table 4 compare the calibration results of the four methods. They are the CAD assembly dimensions between the sensors, the result of EPNP calibration, the calibration result of the 3D-3D [41] calibration method, and the result of using the proposed method. The calibration error Δs is evaluated using the average error of the normalized plane. The evaluation model is as follows.

(24) $${\left[\begin{array}{c} \Delta X\\ \Delta Y\\ 0 \end{array}\right]}_{}=\frac{1}{Z}\left[\begin{array}{c} X\\ Y\\ Z \end{array}\right]-K^{-1}\left[\begin{array}{c} u\\ v\\ 1 \end{array}\right]$$

(25) $$\Delta s=\frac{1}{n}\sum_{i=1}^{n}\sqrt{{\left(\Delta X\right)}^{2}+{\left(\Delta Y\right)}^{2}}$$

where (X,Y,Z) are the spatial coordinates of the feature point in the camera coordinate system, and (u,v) are the coordinates of the feature point in the de-distorted camera image, (ΔX,ΔY) are the errors on the normalized plane after the feature point is re-projected, as shown in Figure 12. K is the camera internal parameter.

**Figure 13:**
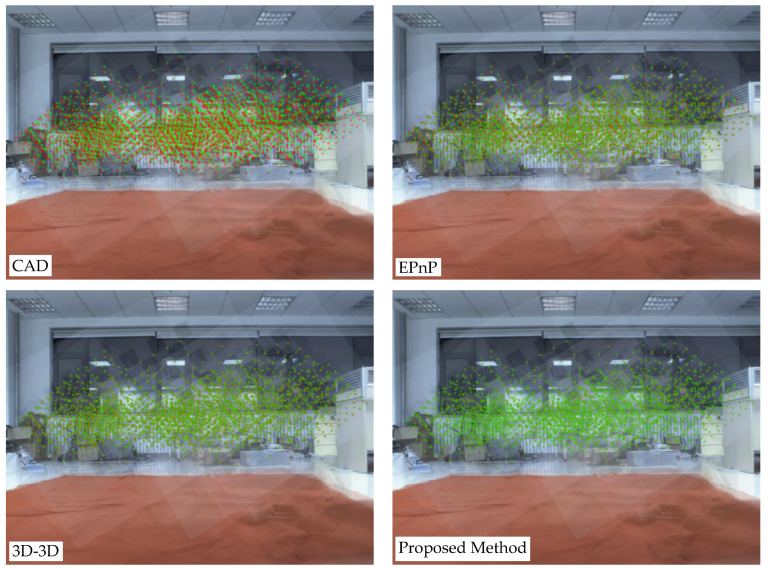
Visual representation of the re-projection error of different methods. Red represents re-projection feature points; green represents feature point pixels.

In order to intuitively observe the accuracy of the calibration results, we first performed a re-projection test on the characteristic corners of the checkerboard, as shown in Figure 13.

As can be seen from Figure 13, using the assembly size of the CAD as a result, a relatively accurate value can be obtained in a certain dimension. However, due to the inevitable assembly error of rotation in the assembly process and the rotation being difficult to measure manually, the rotation error causes a translation error in a certain dimension, and, finally, causes a large error in the entire external parameter. EPnP itself can minimize the error through iteration to optimize the value of the 6-DOF, but the 3d-2d points we obtained through the equipment are often rigid, especially the inaccurate 3d points in the iterative process. It also contributes a large weight, which is not caused by the EPnP algorithm. These inaccurate measurement points eventually lead to inevitable errors in the overall results. In [41], the authors use ArUco code [55,56] to estimate the 3D position of the calibration board’s corner points in the camera coordinate system and use the manually filtered edge point cloud to fit the 3D position of the calibration board’s corner points in the LIDAR coordinate system. Then, the 3D-3D point cloud registration algorithm is used to solve the transformation matrix of the two sets of 3D point clouds and obtain the external parameters of the LIDAR and camera. This method can estimate the accurate external parameters, but the number of original point clouds that can be effectively utilized through manual screening is relatively small. In addition, because there is no correction to the original point cloud, there is still a certain error in the calibration result. Our method first automatically locates the calibration plate position according to the intensity and then fits the virtual feature points. This set of points is obviously inaccurate, including both the measurement error of the radar and the fitting error. However, the advantage of graph optimization is that it can optimize the inaccurate 3D points while optimizing the external parameters and finally estimate the relative pose between the more accurate LIDAR and the camera through joint optimization.

**Figure 14:**
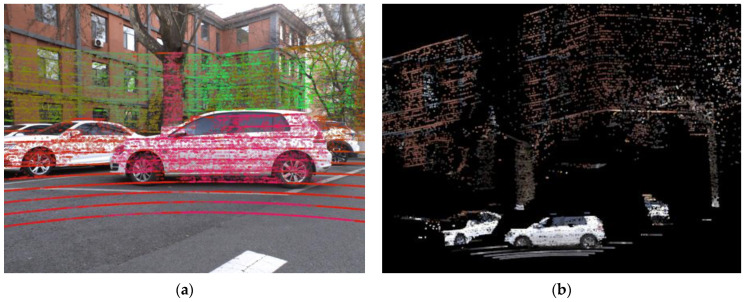
Fusion of image and upsampled point cloud using calibration results. (**a**) Projection from point cloud to image; (**b**) Image color rendering of the point cloud.

We also collected a set of raw data of a complex scene, which contains various objects of different distances and objects with irregular edges. For the same set of raw data and calibration results, Figure 14 shows the effect of using calibration results to fuse the upsampled point cloud and image information, Figure 14a showing the projection from point cloud to image and Figure 14b shows the effect of pixel color rendering of the Lidar point cloud.

In Figure 14a, since the point clouds of different depths are given different colors when projected to the image, it can be seen from the edges between the different colors that the data of the two sensors are well matched. Similarly, since cars, trees, buildings, ground, etc., have high contrast in color, it can be seen from the color point cloud in Figure 14b that the data of the two sensors are well matched.

## 5. Conclusions

Aiming at the problem of external parameter calibration of LIDAR and multi-cameras, this paper proposes an automatic external parameter calibration method based on graph optimization. Through the analysis and experiment of this method, the conclusions are as follows:

(1) Although the data collected by two different types of sensors are different and it is difficult to match the corresponding feature points, we use the reflectivity information of LIDAR to lock and build a virtual calibration board and base it on the virtual calibration board to establish the initial value of the optimization problem. The validity of the initial value is verified by comparing it with the true value of the calibration board size.
(2) Except that the spatial position of the calibration board needs to be manually moved in the data collection stage, the rest of the steps can be completed autonomously, which greatly increases the ease of use of the method. The joint calibration method based on graph optimization can simultaneously optimize the spatial coordinates of the point cloud and the external parameters between multiple sensors, so it is more convenient to calibrate LIDAR and multiple cameras.
(3) All feature points of this method are involved in the optimization calculation, so the accuracy requirements for the initial measured values of the feature points and the initial values of external parameters are relatively low. Through quantitative analysis of different calibration methods, it was found that since the original point cloud is also optimized, the average re-projection error of our calibration result on the normalized plane was 0.161 mm, which is better than the unoptimized calibration method.
(4) From an intuitive qualitative point of view, in complex scenarios, the calibration results can still achieve an accurate fusion of data. The results show that this method can achieve more reliable calibration between lidar and multiple cameras.

## Figures and Tables

**Table 1:** The main parameters of RS-LIDAR-16 LIDAR.

Parameter	Value
Number of channels	16
Effective measuring distance	40 cm~200 m
Precision	± 3 cm
Perspective	Horizontal 360°, vertical −25°~+15°
Angular resolution	Horizontal 0.1°(5 Hz)~0.4°(20 Hz), vertical 0.33°
Rotating speed	300/600/1200 (5/10/20 Hz)
Wavelength	905 nm
Laser emission angle (full angle)	Horizontal 7.4 mRad, vertical 0.7 mRad

**Table 2:** Main parameters of MER-132-43GC-P and ZED 2 cameras.

Parameter	Value	
	MER-132-43GC-P	ZED 2-L
Resolution	1292 × 964	1920 × 1080
Pixel size	3.75 μm × 3.75 μm	2 μm × 2 μm
$f_{x}$	1097.9	1057.47
$f_{y}$	1095.4	1056.79
$c_{x}$	652.383	958.87
$c_{y}$	497.9676	545.144
Distortion coefficient $\left(k_{1},k_{2}\right)$	(−0.0975, 0.0879)	(−0.0437, 0.0122)

**Table 3:** Calibration results of RS-LIDAR-16 and MER-132-43GC-P.

Methods	tx,ty,tz (mm)	roll,pitch,yaw (°)	Re-Projection Error (mm)
CAD	(−3, −93, −95)	(1.5708, 0, 0)	8.874
EPnP [54]	(−0.674, −93.451, −90.725)	(1.57666, 0.00989, −0.01549)	1.748
3D-3D [41]	(−2.112, −92.827, −93.998)	(1.57673, −0.00349, −0.01745)	1.044
Proposed method	(−2.092, −93.018, −94.249)	(1.57666, −0.00343, −0.01650)	0.161

**Table 4:** Calibration results of RS-LIDAR-16 and ZED2-L.

Methods	tx,ty,tz (mm)	roll,pitch,yaw (°)	Re-Projection Error (mm)
CAD	(59.5, 79.5, −5)	(1.5708, 0, 0)	27.68
EPnP [54]	(59.433, 79.623, −2.779)	(1.59471, −0.0154, 0.00543)	1.572
3D-3D [41]	(58.801, 79.402, −5.330)	(1.59470, −0.00601, 0.01286)	1.227
Proposed method	(57.799, 79.398, −6.198)	(1.59471, −0.0061, 0.01283)	0.293
